# Setup in a clinical workflow and impact on radiotherapy routine of an *in vivo* dosimetry procedure with an electronic portal imaging device

**DOI:** 10.1371/journal.pone.0192686

**Published:** 2018-02-12

**Authors:** Jie Li, Angelo Piermattei, Pei Wang, Shengwei Kang, Mingyong Xiao, Bin Tang, Xiongfei Liao, Xin Xin, Mattia Grusio, Lucia Clara Orlandini

**Affiliations:** 1 Department of Radiation Oncology, Sichuan Cancer Hospital, Chengdu, China; 2 UOC Fisica Sanitaria, Fondazione Policlinico Universitario Agostino Gemelli, Rome, Italy; ENEA Centro Ricerche Casaccia, ITALY

## Abstract

High conformal techniques such as intensity-modulated radiation therapy and volumetric-modulated arc therapy are widely used in overloaded radiotherapy departments. *In vivo* dosimetric screening is essential in this environment to avoid important dosimetric errors. This work examines the feasibility of introducing *in vivo* dosimetry (IVD) checks in a radiotherapy routine. The causes of dosimetric disagreements between delivered and planned treatments were identified and corrected during the course of treatment. The efficiency of the corrections performed and the added workload needed for the entire procedure were evaluated.

The IVD procedure was based on an electronic portal imaging device. A total of 3682 IVD tests were performed for 147 patients who underwent head and neck, abdomen, pelvis, breast, and thorax radiotherapy treatments. Two types of indices were evaluated and used to determine if the IVD tests were within tolerance levels: the ratio R between the reconstructed and planned isocentre doses and a transit dosimetry based on the *γ*-analysis of the electronic portal images. The causes of test outside tolerance level were investigated and corrected and IVD test was repeated during subsequent fraction. The time needed for each step of the IVD procedure was registered. Pelvis, abdomen, and head and neck treatments had 10% of tests out of tolerance whereas breast and thorax treatments accounted for up to 25%. The patient setup was the main cause of 90% of the IVD tests out of tolerance and the remaining 10% was due to patient morphological changes. An average time of 42 min per day was sufficient to monitor a daily workload of 60 patients in treatment. This work shows that IVD performed with an electronic portal imaging device is feasible in an overloaded department and enables the timely realignment of the treatment quality indices in order to achieve a patient’s final treatment compliant with the one prescribed.

## Introduction

High conformal techniques such as intensity-modulated radiation therapy (IMRT) and volumetric-modulated arc therapy (VMAT) are widely used for patient treatment and are capable of delivering a high dose to the planning target volume while sparing the surrounding organs at risk. Compared to three-dimensional conformal irradiation techniques, the complexity of the IMRT and VMAT techniques requires every new plan to be monitored prior to delivery as well as monitoring of the dose delivered to the patient during treatment [1]. Inaccuracies in dose delivery may influence the patient outcome for both local tumour control and normal tissue complications due to the steepness of the dose-effect curves [2]. Errors in clinical procedures such as errors in the patient setup, immobilization, or dose variation due to morphological changes [3] cannot be detected by pre-treatment verification or through accurate quality control of the connected machines and medical devices [4, 5]. *In vivo* dosimetry (IVD) tests are performed during the treatment and can detect whether the dose delivered to the patient is within tolerance levels and whether the treatment is dosimetrically reproducible [6, 7]. For these reasons, IVD is one of the major challenges in radiotherapy. In addition, IVD protocols are recommended by different international organizations [8, 9] and, after severe accidents occurred where patients reported several complications, IVD became mandatory in some Western countries [10, 11].

The equipment consists of several linear accelerators (linacs), different treatment planning, and record and verification systems. Each linac supports daily a heavy treatment workload over several shifts. Therefore, patient-specific checks performed while irradiating a phantom-detector combination is time-consuming, and are not applicable; specific protocols have been implemented to verify a percentage of patients according to the technique, linac, and treatment site. Although experiences reported in the literature have indicated that the lack of adequate quality control is responsible for the loss of dosimetric accuracy [12], some authors have reported that patient-specific quality-control usefulness may be questionable [13, 14]. In this environment, IVD checks are strongly recommended to avoid important dosimetric discrepancies. Several researchers [15, 16, 17] have demonstrated the advantages of reconstructing the delivered dose using an amorphous-silicon electronic portal imaging device (EPID), which presents favourable characteristics such as fast image acquisition and high resolution. In addition, IVD methods are still not widely used due to fear of the increased workload required to implement them [7]. Some groups [18, 19, 20, 21] have recently performed patient dose verification using on-treatment EPID transmission images. In the present study, however, the causes of a dosimetric disagreement between the planned and delivered treatment have been analysed and corrected during the course of radiotherapy using an IVD method based on EPID [3, 16, 22, 23, 24]. The efficiency of the corrections performed has been evaluated and the added workload needed for the entire procedure has been registered.

## Materials and methods

### Therapy units and enrolled patients for IVD

A total of 147 patients treated with a photon beam delivered by Elekta Synergy linear accelerators (Elekta, Stockholm, Sweden) available in our department were scheduled for *IVD* with an EPID. All linacs were equipped with an EPID based on panels of aSi detectors (PerkinElmer XRD 1640 AL5, Elekta Crawley, UK) operating as a two-dimensional (2D) photodiode array, an X-ray volumetric imaging (xVi) cone beam computed tomography (CBCT) device, and a HexaPOD robotic couch.

The enrolled patients underwent radiotherapy treatment on different areas of the body (e.g. head and neck (H&N), breast with supraclavicular region, lung, thorax (including lung), abdomen, and pelvis) with a scheduled treatment of more than 20 fractions. VMAT and IMRT treatments were carried out with one of the treatment planning systems (TPSs) available in our department, including the Oncentra Masterplan 4.3, Monaco version 3.0 and 5.0 (Elekta Stockholm, Sweden), Pinnacle 3^TM^ Version 9.10 (Philips Medical Systems, Eindhoven, the Netherlands). The VMAT plans were performed with one or two arcs whereas the IMRT plans had five to nine beams delivered *via* a step-and-shoot technique. At least five IVD tests were scheduled for each patient while 25 to 45 fields were tested for each IMRT patient and five to ten fields were tested for each VMAT patient. Table 1 shows the distribution of the IVD tests versus the TPS used and the adopted technique. The patients were immobilized using personal thermoplastic masks to cover the treatment site and they were allowed to wear thin clothes over the skin for the upper or lower portion of the abdomen and for pelvis and breast treatments. Signs for patient repositioning were drawn onto the mask. CBCT was performed during the first therapy session, referred to in this study as the reference fraction, and then twice a week or before a repeated IVD test after a correction. The couch was moved into the correct position after the CBCT alignment process and the maximum accepted displacements on at least one of the x, y, or z directions following the procedures adopted in our department were ± 5 mm for the pelvis, abdomen, breast, thorax, and ± 3 mm for the H&N.

**Table 1:** Distribution of the 3682 IVD tests versus the TPS used and the treatment technique (VMAT or IMRT) adopted.

	IMRT^a^	VMAT^b^
Oncentra 4.3	1290	-
Monaco 3.0 and 5.0	-	300
Pinnacle 9.10	1675	417

^a^ Intensity modulated radiation therapy

^b^ Volumetric modulated arc therapy

A pre-treatment verification was performed before the beginning of the treatment, comparing the beam x-ray fluence measured by a 2D-array (MatriXX Evolution, IBA Dosimetry GmbH, Schwarzenbruck, Germany) and that computed by the TPS.

The study was reviewed and approved by the Ethics Committee of Sichuan Cancer Hospital.

## IVD procedure

**Fig 1:**
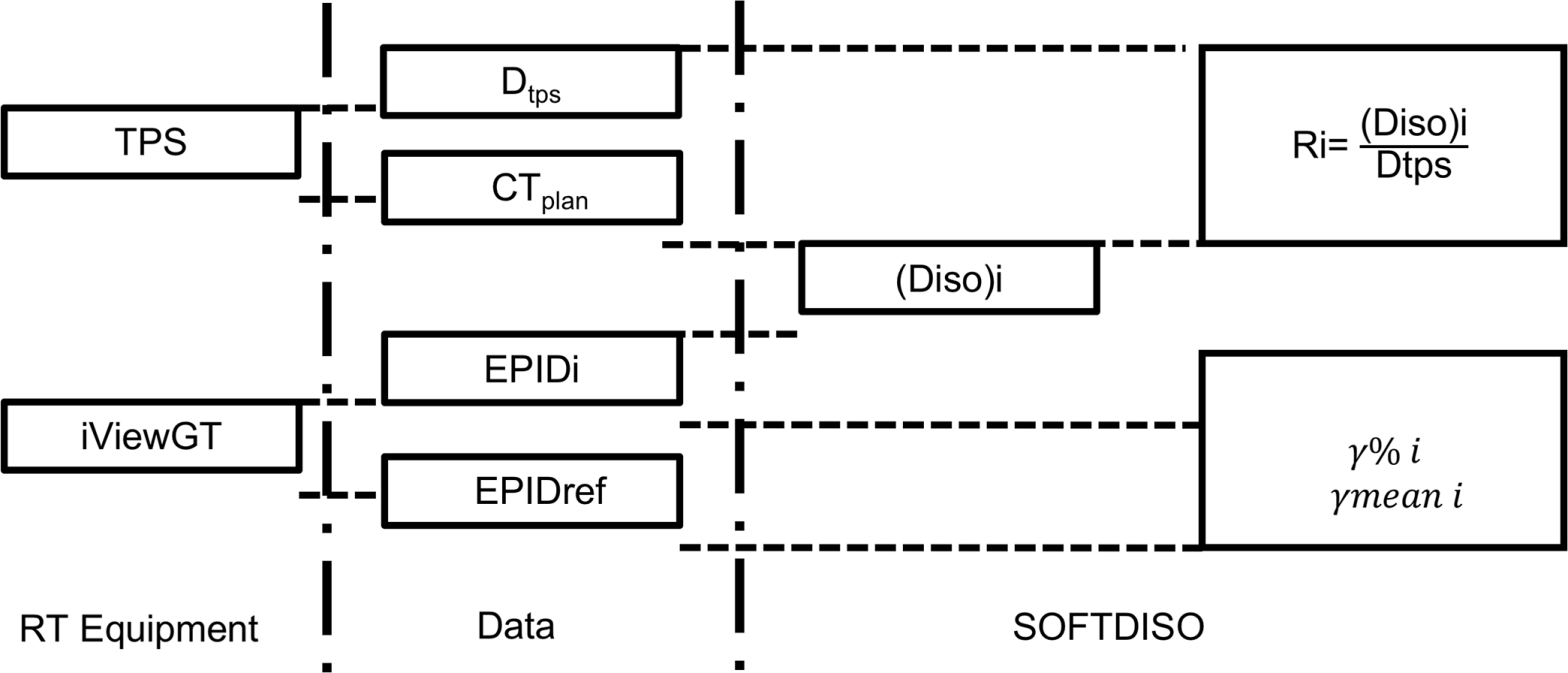
EPID *in vivo* dosimetry workflow. The ratio R = D_iso_/D_tps_ between the reconstructed (D_iso_) and the planned (D_tps_) isocentre dose, the percentage gamma index *γ*%, and the mean value of gamma *γ*_mean_ were evaluated with SOFTDISO using the images of the electronic portal imaging device (EPID) and the data from the TPS and the IViewGT.

The dedicated software SOFTDISO (Best Medical Chianciano, Italy) version 1.24 for EPID *in vivo* dosimetry was used [23, 25]. The software based on a Si-EPID, can be commissioned evaluating the following parameters for each photon energy: the beam quality index TPR^20^_10_ (tissue phantom ratio), the absolute dose (cGy/MU) under reference conditions, a calibration factor k_s_ for the EPID, and its linearity within the range of the monitor unit (MU) that was used [24]. The first two parameters were obtained during the linac commissioning following IAEA TRS-398 [26] while few measurements were performed to characterize the EPID response. The constancy of the EPID calibration factor k_s_ was added to the quality controls of the linac on a weekly basis while the linearity with the MU was inserted in the annual controls [24]. SOFTDISO was connected to the different TPSs to receive DICOM computed tomography (CT) images and the RT plan; the EPID database was used to receive the images acquired during the administration of the treatment. The transfer of the DICOM RT plan and CT scan from the TPS must be performed manually, as must the preparation of the patient in SOFTDISO. The images were transferred to SOFTDISO and automatically evaluated. The software uses a dosimetric method implemented by Piermattei *et al*. [23, 24] that provides two types of IVD tests: the ratio R = D_iso_ / D_tps_ between the reconstructed (D_iso_) and the planned (D_tps_) isocentre dose and a *γ*-analysis obtained between the first EPID image, or reference image (obtained at the reference fraction), and the subsequent images acquired during the course of treatment. The first test is representative of the accuracy of the dose reconstructed at a reference point while the second test is the *γ*-analysis that supplies a transit dosimetry to verify the treatment reproducibility. Both tests supply useful information about the presence of dosimetric errors due to the patient setup, linac output variations, beam interruptions, dose calculations [16, 22], and the presence of patient morphological changes [3, 27]. The *in vivo* EPID-based dosimetry workflow applied in this study is shown in Fig 1.

The index R ratio between the reconstructed and planned isocentre dose is considered in tolerance when 0.95 ≤ R ≤ 1.05. This range takes into account the propagation of the uncertainties of the SOFTDISO reconstruction algorithm for D_iso_ and the TPS reconstruction algorithm for the calculation of D_tps_ [3, 17, 22, 23, 24, 27].

The global *γ*-analysis [28] between the reference EPID and the current images uses two gamma parameters: 1) the EPID percentage signal to agreement ΔS%; and 2) the distance to agreement Δd (mm) [22, 25]. We adopted ΔS% = 3% and Δd = 3 mm for the H&N treatment and ΔS% = 5% and Δd = 5 mm for all other treatment sites. In particular, Δd values were selected to be equal to the maximum displacement acceptable in the clinical practice, while ΔS values were defined by taking into account the presence of heterogeneous tissues, dose gradients, and mobility of the irradiated organs. Following partly the indications for the *γ*-analysis performed for the patient pre-treatment verification, two tolerance levels for the indices were selected: 1) the percentage *γ*-index *γ*% ≥ 90% (i.e. the percentage of points with *γ* < 1 that must be greater than 90%) and 2) the mean *γ* value *γ*_mean_ ≤ 0.4. Therefore, within the EPID irradiated area, a maximum of 10% of the points in disagreement were accepted while the weight of the discrepancy was given by the distribution of *γ* values, which was characterized by a mean value lower than 0.4.

In summary, one test T was defined by the results obtained for the indices R, *γ*% and *γ*_mean_ for each patient and for each beam of the therapy session. In this way, an IVD test warning started when at least one of the three indices was out of tolerance. The aim of the corrective action was to reach values of the three average indices that were in tolerance for each patient (after all the IVD tests), i.e. values of $\bar{R}$ within 5% of, $\bar{\gamma }\%\geq 90\%$, and ${\bar{\gamma }}_{\mathrm{mean}}\leq 0.4$. In particular, a mean value R_beam_ was obtained for each beam by averaging the values of R obtained for this beam on different days; therefore, the value of $\bar{R}$ for a patient was obtained as the average of the mean ratio R_beam_. The indices $\bar{\gamma }$ and ${\bar{\gamma }}_{\mathrm{mean}}$ were obtained with the same modality.

A possible deviation from the planned conditions could be present in the first reference image. The use of pre-treatment verification enables us to exclude deviations in the x-ray fluence. Moreover, the CBCT carried at the reference fraction (and subsequently twice a week), can intercept a deviation from the planned conditions due to morphological changes. In case of a deviation, the reference fraction will be chosen as the subsequent fraction and the reference image is acquired in concomitance with a new CBCT. During the therapy fractions, deviations that can occur (as the unexpected presence of attenuators on the beam) are not taken into consideration in the TPS and are easily intercepted by the indices R and γ.

The time needed for each step of the procedure was registered.

### Management of indices outside tolerance levels

The results of the three indices were displayed for every beam and every fraction on the main screen of the software. In the case of a warning (i.e. when one of the three indices was outside its tolerance level), the cause was investigated first by an experienced medical physicist and subsequently by a radiation oncologist in order to decide the correction to be performed. In this case, another IVD test was performed the following day in concomitance with a new CBCT to verify the effect of the correction.

The R ratios obtained for different fractions and the tolerance threshold (0.95 ≤ R ≤ 1.05) displayed on the main screen of SOFTDISO enable immediate identification of an off-tolerance level (OTL) to investigate a trend that slowly leads to R values beyond acceptable thresholds or simply an acquisition error.

**Fig 2:**
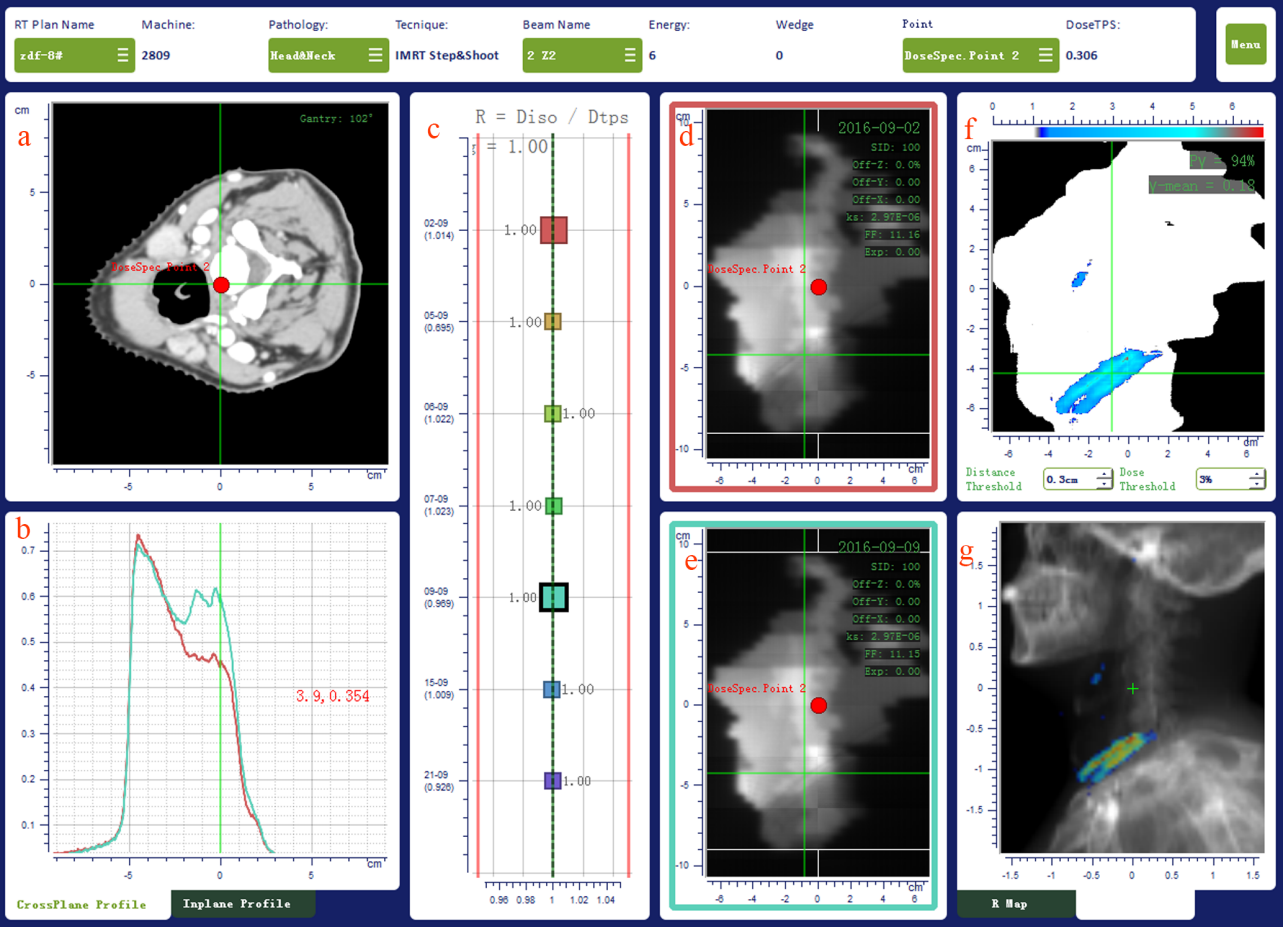
IVD results of a seven beams head and neck IMRT treatment as displayed on the main screen of SOFTDISO. The results for a 102° beam gantry angle of a head and neck IMRT treatment are displayed in (a) patient CT scan containing the isocentre point (red point), (b) EPID signal profiles, (c) R ratio on different days, (d) reference EPID image, (e) current EPID image, (f) map of points with *γ* > 1 (g) map of points with *γ* > 1 on the sagittal DRR image.

The possible reasons of an OTL for the *γ* index can be identified by comparing the inline and crossline signal profiles of the EPID images acquired for different fractions. The *γ-*analysis reported with a map of the points with *γ* > 1 over the digital reconstructed radiography (DRR) in its different projections is also a helpful tool to identify the possible causes of discrepancy. Fig 2 reports an example of IVD test results as displayed on the main screen of SOFTDISO for a 102° beam entry of an H&N IMRT treatment. In this case, the *γ-*analysis of the 5th fraction shows a hot dose region located in correspondence with the patient’s shoulders. The comparison (Fig 2(B)) of the green EPID signal profile of the current fraction with the red profile acquired at the first fraction (reference EPID image), shows a large discrepancy. Moreover, the map of the points with *γ* > 1 (Fig 2(G)) over the digital reconstructed radiography (DRR) indicates a possible shift of the patient’s shoulders. In this case, the patient setup was corrected by a new CBCT and the new IVD test yielded a tolerable value of *γ*% = 98%.

Some IVD tests with OTLs were due to the incorrect positioning of the flat panel, a wrong set up of the acquisition parameters in the iView system, or a lack of synchronization between the image acquisition and delivery (images partially acquired). These cases had high OTLs and were easily identified by observing the results displayed on the main screen of the software (Fig 2(D)). These tests were excluded from the IVD analysis.

From the IVD results, we were able to distinguish two classes of errors referred to as class 1 and class 2. In class 1 errors, OTLs were due to inadequate standard quality controls as defined by the AAPM Task Group 142 [29]. Inadequate controls were the causes of errors in the patient setup (including the accidental presence of attenuators on the beams such as the edge of the beam couch not taken into account by the TPS). In class 2 errors, OTLs were due to patient morphological changes such as tumour shrinkage and loss of patient weight, i.e. all causes that generally require patient morphological controls that were distinguished from the technical and dosimetric controls in this study.

## Results

### IVD tests

The results of our study are reported in terms of the percentage of IVD tests, T%, with indices R, *γ*%, and *γ*_mean_ within tolerance levels and percentage of patients, P%, with mean values of the three indices $\bar{R}$, $\bar{\gamma }\%$, and ${\bar{\gamma }}_{\mathrm{mean}}$ within tolerance levels.

A total of 15% of the EPID images acquired presented artefacts due to errors during the acquisition process and therefore had very high OTLs. These IVD tests are not included in the reported results.

From the analysis of the IVD tests, we can summarize that class 1 errors accounted for 90% of the OTL tests in this study.

The results of 3682 IVD tests carried out for 147 patients undergoing IMRT and VMAT treatments are listed in Tables 2 and 3 in terms of P% and T% with indices within the tolerance levels.

**Table 2:** Percentage of patients P% with indices $\bar{R}$, $\bar{\gamma }$, and ${\bar{\gamma }}_{\mathrm{mean}}$ within tolerance levels for different treatment sites (breast, thorax, abdomen, pelvis, and H&N) and techniques (IMRT and VMAT).

	Breast		Thorax		Abdomen		Pelvis		H&N	
	IMRT	VMAT	IMRT	VMAT	IMRT	VMAT	IMRT	VMAT	IMRT	VMAT
**#Patients^**	8	31	20	6	20	24	12	8	12	6
**P% ($\bar{R}$)**^a^	100	100	100	100	100	100	100	100	100	100
**P%($\bar{\gamma }$%)**^b^	75	100	78	78	100	100	100	100	100	100
**P%**($\bar{\gamma }$_**mean**_**)**^c^	75	100	78	100	100	100	100	100	100	100

^ number of patients evaluated

^a^ percentage of patients with $\bar{R}$ values within tolerance levels

^b^ percentage of patients with, $\bar{\gamma }\%$ values within tolerance levels

^c^ percentage of patients with ${\bar{\gamma }}_{\mathrm{mean}}$ values within tolerance levels

**Table 3:** Percentage of IVD tests T% with indices $\bar{R}$, $\bar{\gamma }$, and ${\bar{\gamma }}_{\mathrm{mean}}$ within tolerance levels for different treatment sites (breast, thorax, abdomen, pelvis, and H&N) and techniques (IMRT and VMAT).

	Breast		Thorax		Abdomen		Pelvis		H&N	
	IMRT	VMAT	IMRT	VMAT	IMRT	VMAT	IMRT	VMAT	IMRT	VMAT
**# tests^**	260	250	749	50	786	238	666	125	504	54
**T% (R)** ^a^	95	88	89	100	89	91	95	100	88	100
**T% (*γ*%)**^b^	75	93	75	76	90	95	95	95	86	90
**T%(*γ***_**mean**_**)**^c^	78	94	80	88	87	95	98	100	92	100

^ number of patients evaluated

^a^ percentage of patients with R values within tolerance levels

^b^ percentage of patients with *γ*% values within tolerance levels

^c^ percentage of patients with *γ*_mean_ values within tolerance levels

The results in Table 2 indicate that for every treatment site and technique adopted, the percentage of patients with values of $\bar{R}$, $\bar{\gamma }\%$, and ${\bar{\gamma }}_{\mathrm{mean}}$ within tolerance levels was 100% for the majority of the treatments performed with the exception of the IMRT treatments of the breast and thorax, which respectively exhibited $P\%(\bar{\gamma }\%)$ and $P\%({\bar{\gamma }}_{\mathrm{mean}})$ values of 75% and 78%, and for the VMAT treatment of the thorax for which $P\%(\bar{\gamma }\%)$ was 78%.

The results in Table 3 show that T% for R values within the tolerance levels is about 90% for the treatment of the abdomen (IMRT and VMAT), breast (VMAT), thorax (IMRT) and H&N (IMRT), and over 95% for the remaining treatment sites and techniques. The values of T% are spread in a wide range: from 75% to 95% and from 78% to 100% for *γ*% and ${\bar{\gamma }}_{\mathrm{mean}}$ indices within tolerance levels, respectively. Breast and thorax were the two treatment sites for which the lowest percentages were obtained, i.e. 75% for breast and thorax (IMRT), 76% for thorax (VMAT).

**Fig 3:**
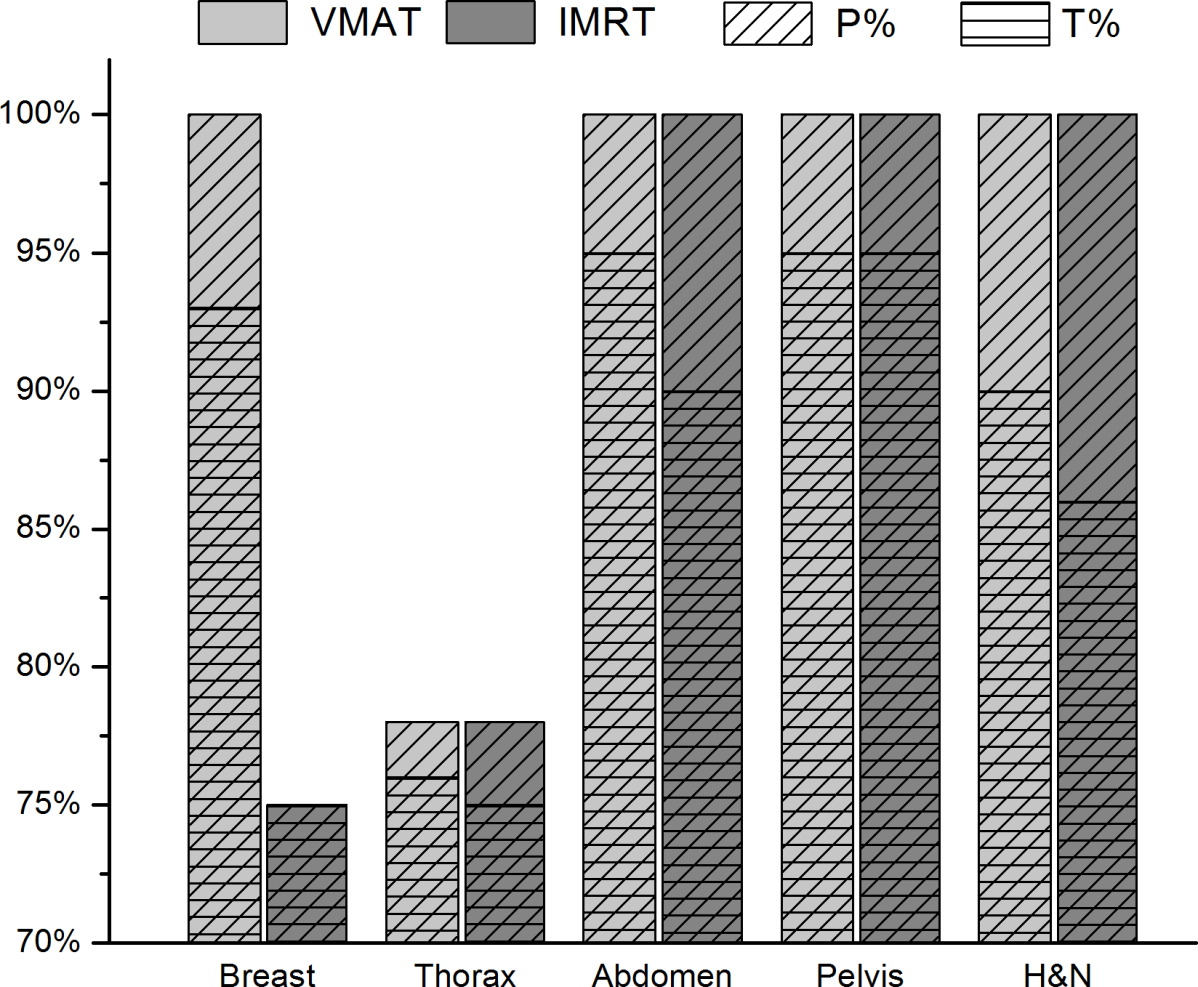
Comparison of the *γ-*analysis results for IMRT and VMAT treatments. The percentage of patients P% (diagonal lines) and the percentage of tests T% (horizontal lines) with gamma indices within tolerance levels are reported for all the treatment sites analysed in this work (breast, thorax, abdomen, pelvis, H&N). The VMAT and IMRT treatments are indicated using light and dark grey colours, respectively.

In general, *γ*% values were clustered around *γ*_mean_. In Fig 3, the results of the *γ-*analysis for IMRT and VMAT treatments are displayed for all the treatment sites. Class 1 errors were particularly important for the breast and thorax areas, for which T% was sensibly lower than for other treatment sites.

### Workload

The daily data acquired and processed by SOFTDISO were evaluated the following morning. The OTL tests were analysed and corrected for the subsequent session that was on the same day for the treatments scheduled in the afternoon or night, and the next day for the treatments scheduled in the morning. Considering a mean workload of 60 patients per day per linac and two IVD checks per week per patient, an average number of 24 patients per day per linac were scheduled for IVD screening. Table 4 shows an analysis of the time required to perform the entire procedure for 24 patients, six of whom recently scheduled. The daily mean values relative to the number of patients tested daily and the relative mean number of tests, the time needed to import the EPID images into SOFTDISO, and the computation time required for the IVD tests were registered. For new patients starting with the IVD procedure, the time to export the patient’s data (DICOM RT plan and the CT scan) from the TPS to SOFTDISO and the time of their commission was added.

**Table 4:** Daily average time of the IVD procedure with 60 patients in treatment. Scheduling two IVD checks per week per patient, an average number of 24 patients per day can be screened with IVD.

P #	T #	IMP (min)	COMP (min)	P* #	T* #	EXP* (min)	IMP* (min)	COMP* (min)	COMM* (min)	Total Time (min)
18	56	9	27(9^)	6	20	6	3	10 (3^)	12	67 (42^)

* refers to the new patients starting with IVD procedure

P#: average number of patients; T#: average number of tests

IMP: average import time (min); time needed to import the EPID images into SOFTDISO

EXP: average export time (min); time needed to export the patient data (CT and RTPLAN) to SOFTDISO

COMP: average computation time; time needed to compute and verify the IVD tests

COMM: average Commissioning time; time (min) needed to commissioning the patient data in SOFTDISO

^ time spent only for the tests out of the tolerance level

The mean total time required for the entire procedure is 67 min, i.e. less than 3 min per patient. Considering that the results of the patients without OTLs were directly stored, the operator can only examine an average of 30% of OTL tests (i.e. those with class 1 and class 2 errors). Thus, the mean computation time was reduced by at least 70% and the overall mean computation time was reduced from 37 to 12 min. Moreover, the overall mean time was reduced from 67 to 42 min (i.e. less than 2 min per patient). The workload for the discussion between medical physicists, radiation oncologists, and therapists pertaining to the OTL tests requires a variable amount of time depending on the complexity of the causes.

## Discussion

The overall number of IVD tests performed for the two different techniques and five treatment sites enable us to formulate some considerations. In the present study, approximately 90% of the OTL tests were due to class 1 errors and were corrected with a systematic check of the patient setup, immobilization system, and alignment, since no errors due to the quality control of the linac, couch, or lasers were found. The rapid computation of the indices enables us to assure an adequate number of IVD tests for each patient. Determining the causes of errors for each OTL index, and adopting the appropriate corrections, the successive IVD tests guaranteed at the end of the treatment course a re-alignment of the average index within the tolerance levels at the end of the treatment course. The remaining 10% of the OTL tests were class 2 errors and, therefore, they were followed individually even for pelvis and abdomen (gas pocket) areas; these errors were adjusted by pushing the patients to follow the indications received during the planning CT pertaining to the daily preparation (e.g. diet, bladder filling, and empty rectum).

As expected, the comparison between Tables 2 and 3 highlighted that the percentage of patients P% with indices within the tolerance levels was in general higher than the percentage of tests T% in tolerance. This shows that the effect of the corrections was evident for all the treatment sites and P% values were equal to 100% with the exception of the breast (IMRT) and thorax (IMRT and VMAT) areas.

The fraction of OTL tests obtained for breasts treated with IMRT was 25% and was partially due to two patients (out of eight) treated with the bolus positioned over the mask and with a mask that did not fit perfectly the body of the patients. Therefore, these patients showed daily a different configuration compared to that of the planning CT for the positioning of the bolus with respect to the treatment site and the air gap between the skin and the mask. The radiation oncologists decided to proceed with new CT scans after three repeated fractions of OTL tests. The effect of the correction was efficient for both patients during the subsequent fractions, even if the mean value of *γ* indices $\bar{\gamma }\%$ and ${\bar{\gamma }}_{\mathrm{mean}}$ were still outside tolerance levels due to the limited number of IVD tests acquired after the correction. However, breast treatments showed a high treatment accuracy considering that 100% of patients for both techniques resulted with an $\bar{R}$ index within tolerance.

In general, the IMRT treatments resulted in a higher percentage of OTL tests compared with those for the VMAT treatments. For an IMRT treatment, each beam corresponds to an IVD test obtained by the EPID image acquired during the delivery. Some beam entries can easily lead to an OTL test if the path of the beam in the patient is varied with respect to the planning configuration, as for the two breast treatment cases described above. For VMAT treatments, the analysed EPID image is obtained by adding the signal of multiple beam entries of the arc; in this way, any inaccuracies of a specific gantry angle can be compensated in the overall arc. Moreover, the bolus automatically created by the treatment planning may lead to a discrepancy between the daily patient setup and that used for the treatment planning. The mould mask performed over the patient’s clothes and not directly on the patient skin can also contribute to a lack of reproducibility in this situation.

For the thorax treatments, the above consideration regarding the patient setup reproducibility remained valid. Two additional contributions to these OTL indices were: 1) the use of a reconstruction point, which can be in a high gradient region; and 2) changes in the patient’s anatomy. As already underlined by Celi et al. [30], the position of the point of interest (in the heterogeneity interface, tongue and groove, and high-dose gradient) plays an important role in case of an observed dose difference. Once these aspects were taken into account, the tests confirmed a correct treatment. However, when P% values less than 100% persisted, this was due to the limited number of tests acquired after the correction.

The abdomen and pelvis treated by IMRT and VMAT resulted in 100% of the patients exhibiting $\bar{R}$, $\bar{\gamma }\%$, and ${\bar{\gamma }}_{\mathrm{mean}}$ indices within tolerance levels. This result confirmed that each patient received a treatment that was compliant with the planned treatment course. The percentage of IVD tests with indices within tolerance levels were less favourable, as a decrement ranging from 5% to 11% was found for the IMRT treatments and a reduction of up to a 9% was found for VMAT treatments. These results were justified by the map of the points with *γ* > 1 over the DRR such as those observed in Fig 2(G); in this case the discrepancy was due to occasional intestine air gap and occasional different filling conditions of the bladder and rectum.

H&N treatments by IMRT and VMAT techniques resulted in 100% of the patients exhibiting $\bar{R}$, $\bar{\gamma }\%$, and ${\bar{\gamma }}_{\mathrm{mean}}$ indices within tolerance levels. The OTL tests occurred mainly because the beam entry had a path within the nasopharyngeal air cavity. For one patient, the loss of weight required a new treatment plan involving a new CT scan and immobilization mask.

Use of CBCT clearly improved the results but it was not able to correct for different patient profile shapes, densities, and depths crossed by the beams. During this start-up period, individual corrections were performed, which were primarily patient-positioning adjustments. In four cases (two breast cases presenting an imperfect fit of the mask positioning, a H&N case associated with weight loss and a thorax case without clear causes), the CBCT required by the OTLs of the IVD tests convinced the radiation oncologist to require an adaptive plan. Following the results obtained in this study, some setup procedures of our department have been revised. In particular, a breast board for the breast and thorax positioning has been adopted instead of the mould mask system. In addition, the bolus is now imaged directly at the planning CT instead of creating it using an automatic tool in the treatment planning system.

There are many important and practical concerns to be addressed for a successful *in vivo* dosimetry procedure such as the identification of an adequate threshold (threshold with clinical relevance) for each parameter followed. Currently, researchers such as Fuangrod et al. [31] are carrying out a statistical process control to identify the most significant threshold that has to be applied in a clinical routine for each treatment site and technique. These evaluations are beyond the scope of this study. Our findings must be understood within the insightful limitation of this research. As reported by other authors[18], the isocentre can be located in a high-dose gradient region or sometimes out of the target and, therefore, it cannot have a clinical significance in some cases. Our study involved patients coming from a single institution and was not validated by a multi-institutional quality assurance program; therefore, the results cannot be generalized. However, the results show that deviations from the initial treatment conditions can arise and that these deviations can be corrected during the course of the treatment if a practical IVD procedure is adopted.

Concerning the accuracy of the actual IVD methods based on EPID, it is important to remember that they are based on the photon fluence reconstruction using the CT scan used for the planning computation. This means that in case of IVD warnings, for example due to an incorrect patient setup or anatomical changes, the reconstructed photon fluence may differ from that used for the dose delivery. Therefore, the 1D, 2D, and 3D IVD tests based on the dose recalculation using the reconstructed photons fluence can present some inaccuracy. However, all the actual procedures can supply useful dose delivery warnings providing indications to prevent errors in the subsequent fractions of the treatment course. Some researchers [32] suggested the use of the CBCT scans to reconstruct the dose in patients using EPID images for the fluence reconstruction, but the CBCT image calibration methods need experimental confirmations and more automatic procedures [33].

In spite of these well-known difficulties, the intention of this work is to continue investigating the feasibility of using calibrated CBCT scans instead of CT scans to assess the dosimetric errors.

## Conclusions

The results obtained for 147 patients highlighted that OTL tests arise during the course of treatment. In this study, over 10% of the IVD tests for the pelvis, abdomen, and H&N treatment for IMRT and up to 25% for the breast and thorax for the VMAT techniques resulted in OTLs. The timely intervention and correction of these errors allows the realignment of the quality indices within the tolerance levels, ensuring that each patient’s final treatment is compliant with the prescribed treatment. Scheduling two IVDs per week per patient, an average time of 42 min per day is sufficient to monitor a daily workload of 60 patients.

In summary, EPID based IVD procedure is a powerful method to monitor the treatment reproducibility and accuracy and to assess the suitability of new techniques and immobilization systems. Its use in a radiotherapy clinical workflow is feasible and has an acceptable added workload.

## Supporting information

S1 File Epid based in vivo dosimetry indices results. The results obtained for R, *γ*%, *γ*_mean_ and registered in the SOFTDISO database are reported in columns G, H and I for every beam tested (column E). The anonymized patient ID, the reference plan, patient pathology, machine ID, are specified in column A, B, C, D respectively.
(XLSX)
